# Academically led clinical trials: challenges and opportunities

**DOI:** 10.1093/annonc/mdv332

**Published:** 2015-08-03

**Authors:** S. Turajlic, J. Larkin, C. Swanton

**Affiliations:** 1 Translational Cancer Therapeutics, The Francis Crick Institute, London; 2 Department of Medical Oncology, The Royal Marsden Hospital, London, UK; 3 University College London Hospitals and Cancer Institute, Huntley Street, London

Highlights from ASCO 2015 demonstrate the impasse we face in solid tumour oncology: the compelling novel immune and targeted therapies are often associated with cost–benefit ratios significantly above the thresholds for reimbursement. This is at least in part a consequence of our incomplete understanding of the mechanisms of response and resistance to these agents. For example, ipilimumab is associated with durable clinical benefit in 15%–20% of unselected advanced melanoma patients (∼£75 000 per patient treated), and while the responses to single-agent targeted therapies such as vemurafenib are higher, they are often relatively short-lived (∼£42 000 per median PFS of 6–7 months). New trial design strategies such as basket and umbrella studies have improved upon patient selection, but have not yielded detailed biological understanding of the drug targets, nor polygenic mechanisms of resistance within or between patients. Academically led studies have the opportunity and the responsibility to prioritize biological insights as trial end points, maximising research gain, increasing patient benefit/safety and ultimately, improving cost-effectiveness. Collection of tumour material is fundamental to these aims but the timing, handling and sample analysis are of critical importance (Figure 1).

Resistance to targeted therapies can be mediated by pre-existing rather than *de novo* alterations. High resolution tracking of cancer cells *in vitro* demonstrated that only 10% of resistant clones arise *de novo* [1], while mathematical models of tumour growth suggest that radiographically detectable lesions harbour at least 10 resistant sub-clones [2]. Thus, comprehensive upfront tumour profiling could anticipate the genetic composition of such clone(s), while taking into account spatial and temporal tumour heterogeneity. Extensive sampling of metastatic sites at autopsy revealed 10 distinct *PTEN* alterations emerging under the selective pressure of PI(3)Kα inhibition [3], and five independent reversion events in a germline *BRCA2* mutant carrier who progressed on olaparib and carboplatin [4]. Distinct mechanisms of BRAF and EGFR inhibitor resistance were detected across multiple metastases within individual patients with melanoma [5] and colorectal cancer [6], respectively.

**Figure 1.**
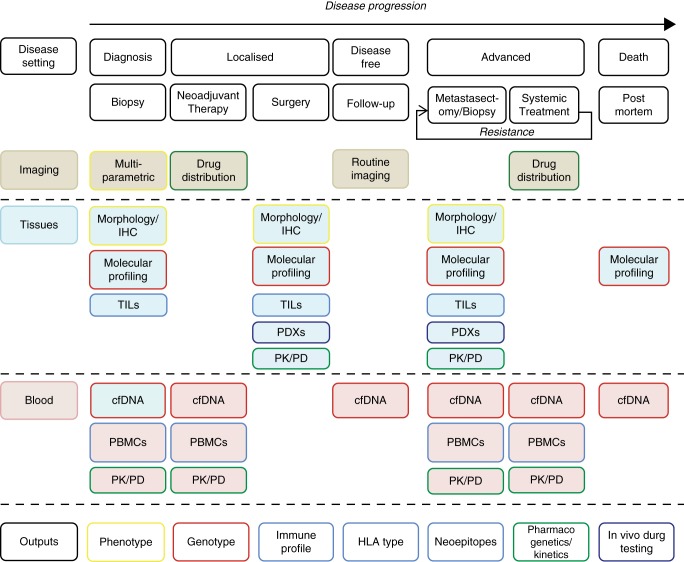
A schematic for biological sample collection throughout the course of disease and treatment. TILs, tumour-infiltrating lymphocytes; cfDNA, cell-free tumour DNA; PBMCs, peripheral mononuclear blood cells; PK, pharmacokinetic; PD, pharmacodynamic; PDX, patient-derived xenograft.

The benefit of combination strategies can be limited by excess toxicity (combined targeting of the PI3K and MAPK pathways [7]), cross-resistance (BRAF and MEK inhibitors in melanoma [8]) and the persistent role of intra-tumour heterogeneity (targeting of the T790M *EGFR* mutation in lung cancer [9]). Informed by pre-clinical models, such as discontinuous dosing in BRAF-mutant melanoma [10], academically led trials can address more finely tuned ways of managing treatment resistance. In colorectal cancer cell-free tumour DNA (cfDNA) shows pulsatile levels of mutant KRAS in response to intermittent EGFR inhibition [11], providing the molecular rationale for re-challenge with targeted therapy. Similar frameworks are required to prospectively evaluate alternative or sequential scheduling as well as the role of cfDNA in tracking tumour progression.

PD-L1 expression, a putative predictive marker for PD1/PDL1 inhibition, is also spatially heterogeneous [12]. Genomic data are a promising alternative biomarker in this area [13]. Mutational data, integrated with HLA typing, and tumour and peripheral T-cell profiling can define individual neo-antigenic repertoires. Academically led studies of immunotherapeutic agents must evaluate the ability of this approach to predict responses, inform immunotherapy/targeted combinations, and ultimately, facilitate adoptive T-cell therapy.

Non-genetic causes of treatment resistance have been largely overlooked but studies that incorporate longitudinal biological sample collection and novel imaging techniques are well placed to examine tumour drug exposure (including heterogeneity of drug distribution [14]) and individual variation in drug metabolising enzymes, receptors, and transporters. Patient-derived xenografts can provide a useful platform for investigating personalised therapy in co-clinical trials [15], but only if robustly characterised and used in the full knowledge of their limitations (e.g. immunosuppressed host, mouse stroma and disparities in tumour burden between mouse and patient).

There clearly are challenges to implementation of such complex studies but they can be overcome through close interdisciplinary work of academic/clinical consortia as illustrated by the Lung TRACERx programme [16], the use of measures such as one-time consent [17], post-mortem studies and stakeholder engagement (patient and public). In summary, we argue for a change of emphasis in drug development from learning little from many patients towards biologically rich clinical studies focussed on gleaning the maximum amount of biological information that might inform drug response and resistance for every patient entered into academic trial protocols.

## disclosure

The authors have declared no conflicts of interest.
